# Di-μ-benzoato-di-μ-ethano­lato-tetra­kis­[μ_3_-5-(hy­droxy­meth­yl)-2-methyl-4-(oxidometh­yl)pyridin-1-ium-3-olato]tetra­kis­[μ_3_-5-(hy­droxy­meth­yl)-2-methyl-4-(oxidometh­yl)pyridin-3-olato]di-μ_3_-oxido-hepta­manganese(II,III) ethanol octa­solvate

**DOI:** 10.1107/S2414314620016430

**Published:** 2021-01-05

**Authors:** Arpita Saha, Clifford W. Padgett, Pierre LeMagueres, Kiana Moncur, Glory Onajobi

**Affiliations:** a Georgia Southern University, 521 C.O.E. Dr., Department of Chemistry and Biochemistry, Statesboro GA 30458, USA; b Rigaku Americas Corporation, 9009 New Trails Drive, The Woodlands TX 77381, USA; Benemérita Universidad Autónoma de Puebla, México

**Keywords:** crystal structure, pyridoxine, manganese, polynuclear complex

## Abstract

Our work in the area of synthesis of polynuclear manganese complexes and their magnetic properties led to the synthesis and crystallization of the title compound, [Mn_7_(C_8_H_9_NO_3_)_4_(C_8_H_10_NO_3_)_4_(C_2_H_5_O)_2_(C_7_H_5_O_2_)_2_O_2_]·8C_2_H_5_OH. Herein, we report the molecular and crystal structure of the title compound, which was synthesized by the reaction of Mn(C_6_H_5_COO)_2_ with pyridoxine (PNH_2_, C_8_H_11_NO_3_) followed by the addition of tetra­methyl­ammonium hydroxide (TMAOH). The core of this centrosymmetric complex is a cage-like structure consisting of six Mn^III^ ions and one Mn^II^ ion bound together through Mn—O bonds. The compound crystallizes in hydrogen-bonded layers formed by O—H⋯N hydrogen bonds involving the aromatic amine group of the ligand PN^2−^ with the neighboring O atoms from the PNH^−^ ligand. The crystal structure has large voids present in which highly disordered solvent mol­ecules (ethanol) sit. A solvent mask was calculated and 181 electrons were found in a volume of 843 Å^3^ in one void per triclinic unit cell. This is consistent with the presence of seven ethanol mol­ecules per formula unit, which accounts for 182 electrons per unit cell. Additionally, one ethanol mol­ecule was found to be ordered in the crystal.

## Abstract 2

The synthesis and structure of a hepta­nuclear cage-like complex, which includes six Mn^III^ ions and one Mn^II^ ion, are presented.

## Graphical abstract

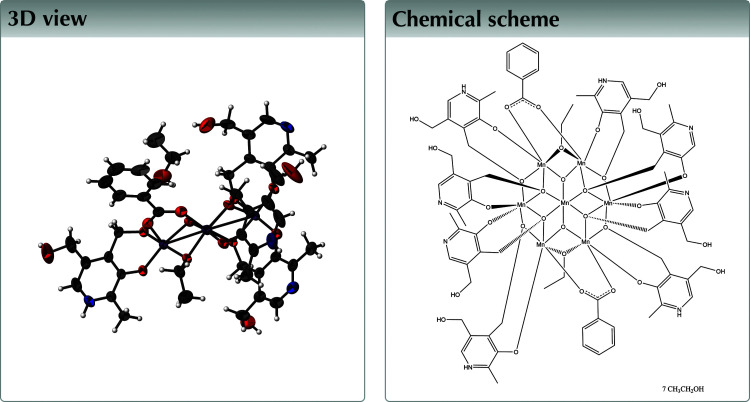

## Structure description

The heptanuclear title compound is [Mn_7_(PN)_4_(PNH)_4_(EtO)_2_(O)_2_(C_6_H_5_CO_2_)_2_]·8(C_2_H_6_O), where PN^2−^ refers to the doubly deprotonated ligand pyridoxine (PNH_2_, C_8_H_11_NO_3_) and PNH^−^ refers to the singly deprotonated ligand. Polynuclear 3*d* metal complexes are known to display aesthetically pleasing structures (Tasiopoulos *et al.*, 2004 ▸), unusual symmetries (Hu *et al.*, 2013 ▸), and unique supra­molecular architectures (Fielden & Cronin, 2005 ▸). Often such polymetallic complexes exhibit magnetic properties (Saha *et al.*, 2011*a* ▸), catalytic properties (Yamada *et al.*, 2015 ▸), optical properties (Aboshyan-Sorgho *et al.*, 2012 ▸) and biological activities (Kuczer *et al.*, 2013 ▸). In order to support the network of three-dimensional polymetallic units, alkoxide-based ligands play an important role since this functionality is an excellent bridging group that fosters higher nuclearity products formation (Saha *et al.*, 2011*b* ▸). Herein, we explore the coordination chemistry of pyridoxine (PNH_2_, IUPAC name: 5-hy­droxy-6-methyl-3,4-pyridinedi­methanol), a water-soluble, naturally occurring vitamer of Vitamin B_6_ involved in the metabolism of all three macronutrients, namely proteins, lipids, and carbohydrates. This ligand plays the pivotal role as a linker (Stouder *et al.*, 2017 ▸).

**Figure 1:**
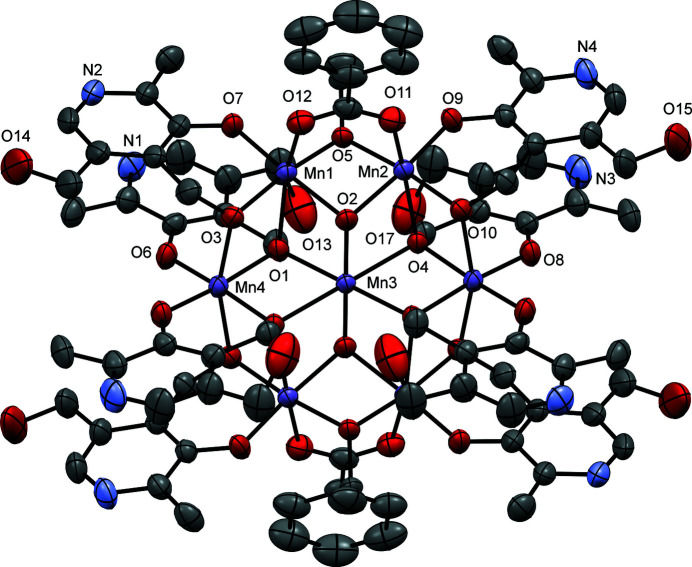
A view of the mol­ecular structure of the title compound, with selected atoms labeled. Hydrogen atoms and solvent mol­ecules are omitted for clarity. Displacement ellipsoids are drawn at the 50% probability level.

**Figure 2:**
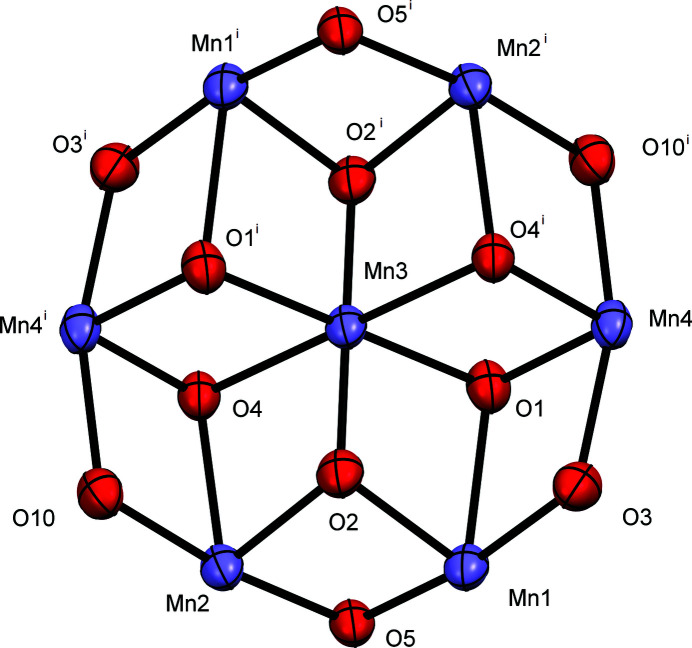
A view of the core of the complex, with the atom labeling. Displacement ellipsoids are drawn at the 50% probability level. [Symmetry code: (i) −*x* + 1, −*y* + 1, −*z* + 1.]

The PNH_2_ ligand is comprised of aliphatic and aromatic alkoxide groups, and those in principle can adopt both bridging and chelating modes while binding with metals. The partially labeled molecular structure of the title compound is shown in Fig. 1 ▸. The core of the centrosymmetric complex (Fig. 2 ▸), is comprised of three triangular Mn_3_ units connected *via* the Mn3 atom at the center of this cage-like structure. The core consists of six Mn^III^ (Mn1, Mn2, Mn4) ions and one Mn^II^ (Mn3) ion. The central Mn3 ion is connected to Mn1 and Mn2 *via* a μ_3_-O oxido ion (O2) and to Mn4 *via* μ_3_-O atoms (O1, O4) coming from the alkoxide arm of a PN^2−^ group that is chelating to Mn4. Apart from that, Mn1 and Mn2 are connected *via* a bridging μ-O atom from the ethoxide group (O5) and a carboxyl­ate group (O11, O12). Mn1 is further connected to Mn4 *via* a bridging μ-O (O3) from the alkoxide arm of a PNH^−^ group and a μ_3_-O atom (O1) from the alkoxide arm of a PN^2−^ group. Similarly, Mn2 is connected to Mn4 *via* a bridging μ-O (O10) from the alkoxide arms of the PNH^−^ group and a μ_3_-O atom (O4) from the alkoxide arm of a PN^2−^ group. The neutral complex is thus comprised of six Mn^III^ ions, one Mn^II^ ion, two oxide ions, two ethoxide ions, two carboxyl­ate ions, four doubly deprotonated, and four singly deprotonated ligands. All Mn ions possess octa­hedral environments. Bond-valence sum (BVS) calculations (Brese & O’Keeffe, 1991 ▸) show that one of the alkoxide arms of all eight PNH_2_ ligands is deprotonated; however, four of the ligands, namely PNH^−^, exist in the zwitterionic form where the aromatic amine functionality is protonated. BVS calculations also confirmed that Mn1, Mn2, and Mn4 are Mn^III^ ions.

**Figure 3:**
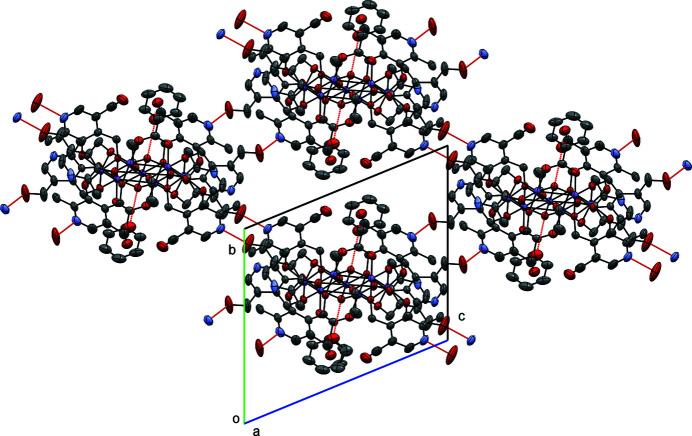
Crystal packing diagram of the title compound viewed along [100]. Hydrogen bonds are colored red.

Inspection of the crystal packing of the complex shows that the Mn_7_ unit relates to its four neighboring units by O—H⋯N hydrogen bonds involving the aromatic amine (N1, N3) group of the ligand PN^2−^ with the neighboring O atoms (O15, O14) from the PNH^−^ ligand (Fig. 3 ▸). In addition to the hydrogen bonds between neighboring molecules, there is also an O—H⋯O hydrogen bond between two OH groups on adjacent ligands (O17, O13). Table 1 ▸ gives details of these hydrogen-bonding inter­actions. The solid-state structural analysis of such complexes can give us valuable insights on potential uses of such materials for catalytic, magnetic and/or biological activity.

The crystal structure has large voids present in which highly disordered solvent mol­ecules (ethanol) sit. A solvent mask was calculated and 181 electrons were found in a volume of 843 Å^3^ in one void per triclinic unit cell. This is consistent with the presence of seven ethanol mol­ecules per formula unit, which accounts for 182 electrons per unit cell. Additionally, one ethanol mol­ecule O16/C42/C43 was found to be ordered in the crystal.

## Synthesis and crystallization

The reaction was carried out in presence of air. To a stirred solution of Mn(C_6_H_5_COO)_2_ (0.17 g, 1.0 mmol) in 12 ml of ethanol, pyridoxine (PNH_2_, 0.10 g, 1.0 mmol) was added at 343 K. The solution turned from pink to light brown after the addition of the PNH_2_, which is an indication of oxidation of Mn^II^ to Mn^III^ by the atmospheric O_2_. After 30 min, TMAOH (0.09 g, 1.0 mmol) was added to the stirred solution. Heating was ceased and the reaction was set to stir for 3 h, after which the dark-brown solution was filtered and set for slow diffusion with Et_2_O. X-ray quality crystals grew after two weeks with a yield of 23%. The crystals were stored in the mother solvent until X-ray study.

## Refinement

Crystal data, data collection and structure refinement details are summarized in Table 2 ▸. Disordered mol­ecules of ethanol were tentatively added to the model. Only one ethanol mol­ecule, with 50% occupancy, refined well without breaking up when anisotropic temperature factors were included, and was kept. The contribution from the other seven disordered ethanol solvent mol­ecules to the structure factors was calculated using the solvent mask tool in *OLEX2* (Dolomanov *et al.*, 2009 ▸).

## Supplementary Material

Crystal structure: contains datablock(s) I. DOI: 10.1107/S2414314620016430/bh4058sup1.cif

Structure factors: contains datablock(s) I. DOI: 10.1107/S2414314620016430/bh4058Isup2.hkl

CCDC reference: 2051338

Additional supporting information:  crystallographic information; 3D view; checkCIF report

## Figures and Tables

**Table 1:** Hydrogen-bond geometry (Å, °)

*D*—H⋯*A*	*D*—H	H⋯*A*	*D*⋯*A*	*D*—H⋯*A*
O14—H14⋯N3^i^	0.84	1.80	2.619 (4)	163
O15—H15⋯N1^ii^	0.84	1.84	2.675 (4)	175
O17—H17⋯O13^iii^	0.84	1.89	2.684 (5)	158

Symmetry codes: (i) 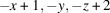 ; (ii) 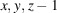 ; (iii) 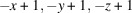 .

**Table 2:** Experimental details

Crystal data	
Chemical formula	[Mn_7_(C_8_H_9_NO_3_)_4_(C_8_H_10_NO_3_)_4_(C_2_H_5_O)_2_(C_7_H_5_O_2_)_2_O_2_]·8C_2_H_6_O
*M*_r_	2449.71
Crystal system, space group	Triclinic, *P* 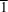
Temperature (K)	100
*a*, *b*, *c* (Å)	12.9774 (5), 14.6762 (7), 16.7750 (6)
α, β, γ (°)	66.578 (4), 77.956 (3), 81.343 (4)
*V* (Å^3^)	2859.0 (2)
*Z*	1
Radiation type	Mo *K*α
μ (mm^−1^)	0.83
Crystal size (mm)	0.09 × 0.08 × 0.06

Data collection	
Diffractometer	Rigaku XtaLAB Synergy, Dualflex, HyPix
Absorption correction	Multi-scan (*CrysAlis PRO*; Rigaku OD, 2020 ▸)
*T*_min_, *T*_max_	0.966, 1.000
No. of measured, independent and observed [*I* > 2σ(*I*)] reflections	34399, 10190, 7884
*R*_int_	0.037
(sin θ/λ)_max_ (Å^−1^)	0.597

Refinement	
*R*[*F* ^2^ > 2σ(*F* ^2^)], *wR*(*F* ^2^), *S*	0.048, 0.141, 1.07
No. of reflections	10190
No. of parameters	621
No. of restraints	9
H-atom treatment	H-atom parameters constrained
Δρ_max_, Δρ_min_ (e Å^−3^)	0.98, −0.28

Computer programs: *CrysAlis PRO* (Rigaku OD, 2020 ▸), *SHELXT* (Sheldrick, 2015*a* ▸), *SHELXL2018/1* (Sheldrick, 2015*b* ▸) and *OLEX2* (Dolomanov *et al.*, 2009 ▸).

## full crystallographic data

### Crystal data

**Table:**

[Mn_7_(C_8_H_9_NO_3_)_4_(C_8_H_10_NO_3_)_4_(C_2_H_5_O)_2_(C_7_H_5_O_2_)_2_O_2_]·8C_2_H_6_O	*Z* = 1
*M**_r_* = 2449.71	*F*(000) = 1274
Triclinic, *P*1	*D*_x_ = 1.423 Mg m^−^^3^
*a* = 12.9774 (5) Å	Mo *K*α radiation, λ = 0.71073 Å
*b* = 14.6762 (7) Å	Cell parameters from 12116 reflections
*c* = 16.7750 (6) Å	θ = 2.6–30.1°
α = 66.578 (4)°	µ = 0.83 mm^−^^1^
β = 77.956 (3)°	*T* = 100 K
γ = 81.343 (4)°	Block, green
*V* = 2859.0 (2) Å^3^	0.09 × 0.08 × 0.06 mm

### Data collection

**Table:**

Rigaku XtaLAB Synergy, Dualflex, HyPix diffractometer	10190 independent reflections
Radiation source: micro-focus sealed X-ray tube, PhotonJet (Mo) X-ray Source	7884 reflections with *I* > 2σ(*I*)
Mirror monochromator	*R*_int_ = 0.037
Detector resolution: 10.0000 pixels mm^-1^	θ_max_ = 25.1°, θ_min_ = 2.5°
ω scans	*h* = −15→15
Absorption correction: multi-scan (CrysAlisPro; Rigaku OD, 2020)	*k* = −17→17
*T*_min_ = 0.966, *T*_max_ = 1.000	*l* = −20→20
34399 measured reflections	

### Refinement

**Table:**

Refinement on *F*^2^	Primary atom site location: dual
Least-squares matrix: full	Secondary atom site location: difference Fourier map
*R*[*F*^2^ > 2σ(*F*^2^)] = 0.048	Hydrogen site location: inferred from neighbouring sites
*wR*(*F*^2^) = 0.141	H-atom parameters constrained
*S* = 1.07	*w* = 1/[σ^2^(*F*_o_^2^) + (0.0847*P*)^2^ + 0.6047*P*] where *P* = (*F*_o_^2^ + 2*F*_c_^2^)/3
10190 reflections	(Δ/σ)_max_ = 0.001
621 parameters	Δρ_max_ = 0.98 e Å^−^^3^
9 restraints	Δρ_min_ = −0.28 e Å^−^^3^

### Special details

**Table:**

Refinement. Hydrogen atoms were attached *via* the riding model at calculated positions using suitable HFIX commands.

### Fractional atomic coordinates and isotropic or equivalent isotropic displacement parameters (Å^2^)

**Table:**

	*x*	*y*	*z*	*U*_iso_*/*U*_eq_	Occ. (<1)
Mn1	0.72295 (3)	0.42295 (3)	0.56146 (3)	0.03246 (13)	
Mn2	0.72328 (3)	0.52363 (3)	0.37463 (3)	0.03223 (13)	
Mn3	0.500000	0.500000	0.500000	0.02829 (15)	
Mn4	0.49800 (4)	0.39857 (3)	0.70324 (3)	0.03243 (13)	
O1	0.55540 (16)	0.51184 (14)	0.60120 (12)	0.0335 (5)	
O2	0.63608 (16)	0.43484 (15)	0.47679 (12)	0.0346 (5)	
O3	0.64800 (16)	0.32378 (15)	0.65648 (13)	0.0377 (5)	
O4	0.43587 (16)	0.37094 (14)	0.62110 (12)	0.0317 (4)	
O5	0.77496 (16)	0.54310 (15)	0.46695 (12)	0.0340 (5)	
O6	0.54308 (18)	0.43981 (16)	0.78180 (13)	0.0427 (5)	
O7	0.80492 (17)	0.43486 (16)	0.63726 (13)	0.0399 (5)	
O8	0.45964 (18)	0.27999 (16)	0.79730 (13)	0.0429 (5)	
O9	0.80638 (17)	0.62354 (16)	0.28792 (12)	0.0401 (5)	
O10	0.65480 (16)	0.51228 (16)	0.29100 (12)	0.0365 (5)	
O11	0.84197 (18)	0.40553 (17)	0.37249 (14)	0.0450 (5)	
O12	0.84048 (18)	0.32516 (16)	0.51759 (14)	0.0446 (5)	
O13	0.5745 (3)	0.8308 (2)	0.5748 (2)	0.0938 (11)	
H13	0.563029	0.867061	0.604331	0.141*	
O14	0.7656 (3)	0.0844 (3)	0.9805 (2)	0.1267 (17)	
H14	0.722079	0.044696	1.017122	0.190*	
O15	0.7882 (3)	0.6430 (4)	−0.07481 (18)	0.1081 (14)	
H15	0.752050	0.628887	−0.103888	0.162*	
O17	0.4330 (3)	0.0810 (3)	0.5982 (2)	0.0919 (11)	
H17	0.423053	0.094953	0.546618	0.138*	
N1	0.6795 (3)	0.6054 (2)	0.82394 (19)	0.0565 (8)	
N2	0.8660 (2)	0.3582 (3)	0.85144 (18)	0.0572 (8)	
H2	0.898645	0.380528	0.880277	0.069*	
N3	0.3404 (3)	0.0499 (2)	0.8848 (2)	0.0625 (9)	
N4	0.8846 (3)	0.7742 (2)	0.06429 (17)	0.0541 (8)	
H4	0.919064	0.827978	0.033777	0.065*	
C1	0.5477 (3)	0.6057 (2)	0.6102 (2)	0.0437 (8)	
H1A	0.472136	0.628671	0.620333	0.052*	
H1B	0.581389	0.655189	0.554310	0.052*	
C2	0.5985 (3)	0.6023 (2)	0.68398 (19)	0.0398 (7)	
C3	0.5897 (2)	0.5214 (2)	0.7651 (2)	0.0383 (7)	
C4	0.6299 (3)	0.5275 (3)	0.8349 (2)	0.0453 (8)	
C5	0.6900 (4)	0.6800 (3)	0.7461 (3)	0.0668 (12)	
H5	0.726298	0.734859	0.739336	0.080*	
C6	0.6519 (3)	0.6829 (3)	0.6746 (2)	0.0541 (9)	
C7	0.6150 (3)	0.4451 (3)	0.9227 (2)	0.0521 (9)	
H7A	0.657552	0.453886	0.960656	0.078*	
H7B	0.637445	0.381370	0.915995	0.078*	
H7C	0.540275	0.445717	0.949300	0.078*	
C8	0.6669 (4)	0.7730 (3)	0.5903 (3)	0.0711 (13)	
H8A	0.719651	0.813228	0.593372	0.085*	
H8B	0.694921	0.751164	0.540623	0.085*	
C9	0.7070 (3)	0.2479 (2)	0.7162 (2)	0.0480 (8)	
H9A	0.759715	0.213800	0.683475	0.058*	
H9B	0.658742	0.198088	0.759588	0.058*	
C10	0.7632 (3)	0.2870 (2)	0.7647 (2)	0.0438 (8)	
C11	0.7743 (3)	0.2318 (3)	0.8526 (2)	0.0579 (10)	
C12	0.8258 (3)	0.2700 (3)	0.8944 (2)	0.0657 (12)	
H12	0.832882	0.233481	0.954365	0.079*	
C13	0.8599 (3)	0.4152 (3)	0.7668 (2)	0.0445 (8)	
C14	0.8080 (2)	0.3788 (2)	0.72120 (19)	0.0401 (7)	
C15	0.7288 (3)	0.1302 (4)	0.9029 (3)	0.0804 (15)	
H15A	0.650705	0.138997	0.914161	0.096*	
H15B	0.749629	0.088584	0.867154	0.096*	
C16	0.9105 (3)	0.5112 (3)	0.7254 (2)	0.0543 (9)	
H16A	0.900561	0.543147	0.768119	0.082*	
H16B	0.878194	0.554992	0.674076	0.082*	
H16C	0.986260	0.498703	0.706714	0.082*	
C17	0.4377 (3)	0.2715 (2)	0.62780 (19)	0.0396 (7)	
H17A	0.510846	0.250131	0.607072	0.048*	
H17B	0.392631	0.270284	0.587405	0.048*	
C18	0.4011 (3)	0.1963 (2)	0.7189 (2)	0.0404 (7)	
C19	0.4165 (2)	0.2055 (2)	0.7954 (2)	0.0385 (7)	
C20	0.3851 (3)	0.1288 (2)	0.8786 (2)	0.0470 (8)	
C21	0.3266 (4)	0.0416 (3)	0.8115 (3)	0.0703 (12)	
H21	0.295627	−0.015756	0.817061	0.084*	
C22	0.3544 (3)	0.1115 (3)	0.7281 (2)	0.0552 (10)	
C23	0.4023 (3)	0.1353 (3)	0.9612 (2)	0.0558 (10)	
H23A	0.474186	0.108487	0.971879	0.084*	
H23B	0.351217	0.096644	1.010739	0.084*	
H23C	0.392797	0.205080	0.955318	0.084*	
C24	0.3357 (4)	0.0933 (3)	0.6510 (3)	0.0709 (13)	
H24A	0.296834	0.032815	0.672004	0.085*	
H24B	0.291910	0.150337	0.615491	0.085*	
C25	0.8829 (2)	0.5660 (3)	0.4548 (2)	0.0438 (8)	
H25A	0.922119	0.560551	0.399362	0.053*	
H25B	0.917118	0.516849	0.503944	0.053*	
C26	0.8890 (3)	0.6662 (3)	0.4515 (3)	0.0716 (12)	
H26A	0.849209	0.714489	0.406701	0.107*	
H26B	0.962998	0.682037	0.436342	0.107*	
H26C	0.858678	0.669216	0.509054	0.107*	
C27	0.8156 (2)	0.6543 (2)	0.20096 (18)	0.0347 (7)	
C28	0.8713 (3)	0.7392 (2)	0.1522 (2)	0.0422 (8)	
C29	0.8469 (3)	0.7298 (3)	0.0209 (2)	0.0587 (10)	
H29	0.859961	0.755807	−0.041632	0.070*	
C30	0.7901 (3)	0.6477 (3)	0.0657 (2)	0.0488 (9)	
C31	0.7733 (2)	0.6081 (2)	0.15795 (19)	0.0372 (7)	
C32	0.7148 (3)	0.5151 (3)	0.20907 (19)	0.0421 (8)	
H32A	0.666870	0.509853	0.172562	0.050*	
H32B	0.766723	0.456435	0.219945	0.050*	
C33	0.9166 (3)	0.7920 (3)	0.1953 (2)	0.0531 (9)	
H33A	0.947473	0.852197	0.150287	0.080*	
H33B	0.971503	0.748021	0.227416	0.080*	
H33C	0.860547	0.810577	0.236486	0.080*	
C34	0.7481 (3)	0.5992 (4)	0.0152 (2)	0.0709 (13)	
H34A	0.769456	0.526970	0.037316	0.085*	
H34B	0.669927	0.607750	0.024434	0.085*	
C35	0.8716 (3)	0.3350 (2)	0.4376 (2)	0.0429 (8)	
C36	0.9487 (3)	0.2559 (3)	0.4202 (3)	0.0499 (9)	
C37	0.9834 (3)	0.2609 (3)	0.3344 (3)	0.0599 (10)	
H37	0.957347	0.314580	0.286968	0.072*	
C38	1.0550 (4)	0.1889 (4)	0.3176 (3)	0.0740 (12)	
H38	1.077694	0.193154	0.258671	0.089*	
C39	1.0935 (4)	0.1118 (4)	0.3846 (4)	0.0821 (14)	
H39	1.142783	0.062191	0.372524	0.099*	
C40	1.0608 (4)	0.1056 (3)	0.4700 (4)	0.0740 (13)	
H40	1.088196	0.052042	0.516736	0.089*	
C41	0.9875 (3)	0.1777 (3)	0.4883 (3)	0.0574 (10)	
H41	0.964559	0.172784	0.547368	0.069*	
O16	0.6762 (5)	0.2505 (4)	0.4357 (4)	0.0752 (16)	0.5
H16	0.642396	0.290669	0.457791	0.113*	0.5
C42	0.6885 (6)	0.1580 (6)	0.5016 (6)	0.0623 (17)	0.5
H42A	0.623729	0.143483	0.546420	0.075*	0.5
H42B	0.748686	0.154590	0.530817	0.075*	0.5
C43	0.7077 (7)	0.0916 (7)	0.4594 (7)	0.081 (2)	0.5
H43A	0.673340	0.118745	0.407039	0.121*	0.5
H43B	0.679244	0.028034	0.499495	0.121*	0.5
H43C	0.783989	0.081056	0.441767	0.121*	0.5

### Atomic displacement parameters (Å^2^)

**Table:**

	*U*^11^	*U*^22^	*U*^33^	*U*^12^	*U*^13^	*U*^23^
Mn1	0.0324 (3)	0.0366 (3)	0.0289 (2)	−0.0128 (2)	−0.00724 (18)	−0.00816 (18)
Mn2	0.0324 (3)	0.0407 (3)	0.0269 (2)	−0.0137 (2)	−0.00393 (18)	−0.01295 (19)
Mn3	0.0290 (3)	0.0337 (3)	0.0242 (3)	−0.0124 (3)	−0.0051 (2)	−0.0093 (2)
Mn4	0.0368 (3)	0.0378 (3)	0.0241 (2)	−0.0140 (2)	−0.00685 (18)	−0.00830 (18)
O1	0.0416 (12)	0.0341 (11)	0.0270 (10)	−0.0156 (9)	−0.0040 (8)	−0.0099 (8)
O2	0.0350 (12)	0.0402 (11)	0.0317 (10)	−0.0157 (9)	−0.0073 (8)	−0.0114 (9)
O3	0.0363 (12)	0.0360 (11)	0.0363 (11)	−0.0132 (9)	−0.0077 (9)	−0.0043 (9)
O4	0.0361 (11)	0.0345 (11)	0.0254 (9)	−0.0145 (9)	−0.0056 (8)	−0.0078 (8)
O5	0.0333 (11)	0.0419 (11)	0.0284 (10)	−0.0166 (9)	−0.0042 (8)	−0.0107 (8)
O6	0.0535 (14)	0.0479 (13)	0.0298 (11)	−0.0168 (11)	−0.0139 (9)	−0.0098 (9)
O7	0.0442 (13)	0.0449 (12)	0.0305 (11)	−0.0199 (10)	−0.0099 (9)	−0.0065 (9)
O8	0.0528 (14)	0.0455 (13)	0.0274 (10)	−0.0202 (11)	−0.0076 (9)	−0.0044 (9)
O9	0.0459 (13)	0.0528 (13)	0.0274 (10)	−0.0247 (11)	−0.0002 (9)	−0.0169 (9)
O10	0.0391 (12)	0.0462 (12)	0.0308 (10)	−0.0153 (10)	−0.0038 (8)	−0.0180 (9)
O11	0.0438 (13)	0.0499 (14)	0.0467 (13)	−0.0056 (11)	−0.0058 (10)	−0.0241 (11)
O12	0.0411 (13)	0.0443 (13)	0.0506 (14)	−0.0055 (10)	−0.0090 (10)	−0.0187 (10)
O13	0.140 (3)	0.0525 (18)	0.074 (2)	0.000 (2)	0.001 (2)	−0.0191 (15)
O14	0.099 (3)	0.139 (3)	0.078 (2)	−0.053 (2)	−0.034 (2)	0.050 (2)
O15	0.113 (3)	0.189 (4)	0.0413 (16)	−0.080 (3)	−0.0050 (16)	−0.044 (2)
O17	0.133 (3)	0.082 (2)	0.083 (2)	0.008 (2)	−0.040 (2)	−0.050 (2)
N1	0.067 (2)	0.069 (2)	0.0511 (17)	−0.0224 (17)	−0.0145 (15)	−0.0322 (16)
N2	0.0421 (17)	0.093 (2)	0.0352 (15)	−0.0091 (16)	−0.0118 (12)	−0.0189 (16)
N3	0.082 (2)	0.0445 (17)	0.0467 (18)	−0.0233 (17)	−0.0100 (16)	0.0036 (14)
N4	0.070 (2)	0.0525 (18)	0.0327 (15)	−0.0244 (16)	−0.0011 (13)	−0.0058 (13)
C1	0.062 (2)	0.0350 (17)	0.0387 (17)	−0.0155 (15)	−0.0122 (15)	−0.0127 (13)
C2	0.0406 (18)	0.0474 (18)	0.0366 (16)	−0.0100 (15)	−0.0038 (13)	−0.0200 (14)
C3	0.0354 (17)	0.0479 (18)	0.0403 (17)	−0.0094 (14)	−0.0082 (13)	−0.0227 (14)
C4	0.048 (2)	0.060 (2)	0.0373 (17)	−0.0073 (16)	−0.0081 (14)	−0.0261 (15)
C5	0.093 (3)	0.064 (2)	0.057 (2)	−0.037 (2)	−0.017 (2)	−0.025 (2)
C6	0.065 (2)	0.062 (2)	0.0480 (19)	−0.0258 (19)	−0.0099 (17)	−0.0268 (17)
C7	0.061 (2)	0.065 (2)	0.0404 (18)	−0.0041 (18)	−0.0170 (16)	−0.0265 (17)
C8	0.103 (4)	0.062 (3)	0.059 (2)	−0.045 (3)	−0.015 (2)	−0.021 (2)
C9	0.047 (2)	0.0394 (18)	0.0468 (18)	−0.0080 (15)	−0.0133 (15)	−0.0004 (14)
C10	0.0345 (18)	0.0463 (19)	0.0381 (17)	−0.0076 (14)	−0.0100 (13)	0.0005 (14)
C11	0.037 (2)	0.072 (3)	0.0436 (19)	−0.0102 (18)	−0.0120 (15)	0.0050 (17)
C12	0.043 (2)	0.100 (3)	0.0308 (18)	−0.007 (2)	−0.0095 (15)	0.0026 (19)
C13	0.0352 (18)	0.065 (2)	0.0342 (17)	−0.0054 (16)	−0.0066 (13)	−0.0187 (15)
C14	0.0334 (17)	0.0497 (19)	0.0327 (16)	−0.0053 (14)	−0.0083 (13)	−0.0088 (14)
C15	0.056 (3)	0.098 (3)	0.052 (2)	−0.020 (2)	−0.0154 (19)	0.017 (2)
C16	0.057 (2)	0.068 (2)	0.054 (2)	−0.0170 (19)	−0.0147 (17)	−0.0313 (18)
C17	0.0459 (19)	0.0403 (17)	0.0372 (16)	−0.0132 (14)	−0.0083 (14)	−0.0152 (13)
C18	0.0438 (19)	0.0339 (16)	0.0405 (17)	−0.0100 (14)	−0.0091 (14)	−0.0074 (13)
C19	0.0384 (18)	0.0336 (16)	0.0374 (16)	−0.0080 (14)	−0.0057 (13)	−0.0056 (13)
C20	0.051 (2)	0.0399 (18)	0.0399 (18)	−0.0065 (16)	−0.0060 (15)	−0.0043 (14)
C21	0.101 (4)	0.042 (2)	0.063 (3)	−0.035 (2)	−0.021 (2)	0.0003 (18)
C22	0.076 (3)	0.0410 (19)	0.049 (2)	−0.0205 (18)	−0.0168 (18)	−0.0080 (15)
C23	0.071 (3)	0.047 (2)	0.0344 (17)	−0.0094 (18)	−0.0035 (16)	−0.0002 (15)
C24	0.105 (4)	0.047 (2)	0.065 (3)	−0.034 (2)	−0.021 (2)	−0.0123 (19)
C25	0.0346 (18)	0.062 (2)	0.0352 (16)	−0.0206 (16)	−0.0073 (13)	−0.0121 (15)
C26	0.058 (3)	0.090 (3)	0.090 (3)	−0.035 (2)	−0.002 (2)	−0.052 (3)
C27	0.0361 (17)	0.0407 (17)	0.0291 (15)	−0.0071 (13)	−0.0024 (12)	−0.0150 (12)
C28	0.046 (2)	0.0444 (18)	0.0360 (17)	−0.0111 (15)	0.0003 (14)	−0.0156 (14)
C29	0.068 (3)	0.074 (3)	0.0275 (17)	−0.019 (2)	−0.0058 (16)	−0.0087 (16)
C30	0.052 (2)	0.068 (2)	0.0324 (17)	−0.0186 (18)	−0.0068 (14)	−0.0208 (16)
C31	0.0332 (17)	0.0505 (19)	0.0324 (15)	−0.0076 (14)	−0.0028 (12)	−0.0202 (14)
C32	0.048 (2)	0.053 (2)	0.0351 (16)	−0.0161 (16)	−0.0026 (14)	−0.0251 (14)
C33	0.064 (2)	0.052 (2)	0.0493 (19)	−0.0304 (18)	0.0074 (17)	−0.0244 (16)
C34	0.067 (3)	0.116 (4)	0.0329 (18)	−0.039 (3)	−0.0045 (17)	−0.023 (2)
C35	0.0377 (18)	0.0455 (19)	0.052 (2)	−0.0145 (15)	−0.0017 (15)	−0.0246 (16)
C36	0.041 (2)	0.0444 (19)	0.073 (2)	−0.0172 (16)	−0.0055 (17)	−0.0277 (18)
C37	0.050 (2)	0.060 (2)	0.082 (3)	−0.0166 (19)	0.0028 (19)	−0.041 (2)
C38	0.068 (3)	0.071 (3)	0.098 (3)	−0.010 (2)	0.003 (2)	−0.054 (3)
C39	0.066 (3)	0.070 (3)	0.128 (4)	−0.003 (2)	−0.006 (3)	−0.062 (3)
C40	0.060 (3)	0.049 (2)	0.124 (4)	−0.003 (2)	−0.028 (3)	−0.038 (3)
C41	0.047 (2)	0.052 (2)	0.083 (3)	−0.0119 (18)	−0.0090 (19)	−0.033 (2)
O16	0.061 (4)	0.071 (3)	0.103 (4)	−0.008 (3)	−0.034 (3)	−0.032 (3)
C42	0.026 (3)	0.066 (3)	0.096 (5)	−0.013 (3)	−0.005 (3)	−0.031 (3)
C43	0.054 (5)	0.077 (4)	0.123 (7)	0.017 (4)	−0.031 (5)	−0.050 (5)

### Geometric parameters (Å, º)

**Table:**

Mn1—Mn2	2.8844 (6)	C8—H8B	0.9900
Mn1—Mn3	3.1695 (5)	C9—H9A	0.9900
Mn1—O1	2.471 (2)	C9—H9B	0.9900
Mn1—O2	1.9315 (19)	C9—C10	1.506 (5)
Mn1—O3	1.8815 (19)	C10—C11	1.398 (4)
Mn1—O5	1.9410 (19)	C10—C14	1.400 (5)
Mn1—O7	1.891 (2)	C11—C12	1.369 (6)
Mn1—O12	2.154 (2)	C11—C15	1.529 (6)
Mn2—Mn3	3.1810 (4)	C12—H12	0.9500
Mn2—O2	1.9428 (19)	C13—C14	1.405 (5)
Mn2—O4^i^	2.394 (2)	C13—C16	1.486 (5)
Mn2—O5	1.9422 (19)	C15—H15A	0.9900
Mn2—O9	1.8853 (19)	C15—H15B	0.9900
Mn2—O10	1.881 (2)	C16—H16A	0.9800
Mn2—O11	2.145 (2)	C16—H16B	0.9800
Mn3—Mn4^i^	3.1274 (4)	C16—H16C	0.9800
Mn3—Mn4	3.1274 (4)	C17—H17A	0.9900
Mn3—O1	2.0519 (19)	C17—H17B	0.9900
Mn3—O1^i^	2.0518 (19)	C17—C18	1.515 (4)
Mn3—O2	1.920 (2)	C18—C19	1.400 (4)
Mn3—O2^i^	1.920 (2)	C18—C22	1.404 (5)
Mn3—O4^i^	2.2620 (18)	C19—C20	1.426 (4)
Mn3—O4	2.2620 (18)	C20—C23	1.490 (5)
Mn4—O1	1.9563 (18)	C21—H21	0.9500
Mn4—O3	2.239 (2)	C21—C22	1.379 (5)
Mn4—O4	1.9286 (19)	C22—C24	1.492 (5)
Mn4—O6	1.873 (2)	C23—H23A	0.9800
Mn4—O8	1.875 (2)	C23—H23B	0.9800
Mn4—O10^i^	2.212 (2)	C23—H23C	0.9800
O1—C1	1.432 (4)	C24—H24A	0.9900
O3—C9	1.417 (4)	C24—H24B	0.9900
O4—C17	1.415 (4)	C25—H25A	0.9900
O5—C25	1.445 (3)	C25—H25B	0.9900
O6—C3	1.327 (4)	C25—C26	1.462 (5)
O7—C14	1.324 (3)	C26—H26A	0.9800
O8—C19	1.315 (4)	C26—H26B	0.9800
O9—C27	1.329 (3)	C26—H26C	0.9800
O10—C32	1.420 (3)	C27—C28	1.400 (4)
O11—C35	1.252 (4)	C27—C31	1.396 (4)
O12—C35	1.274 (4)	C28—C33	1.493 (5)
O13—H13	0.8400	C29—H29	0.9500
O13—C8	1.368 (6)	C29—C30	1.370 (5)
O14—H14	0.8400	C30—C31	1.400 (4)
O14—C15	1.356 (5)	C30—C34	1.520 (5)
O15—H15	0.8400	C31—C32	1.511 (4)
O15—C34	1.402 (4)	C32—H32A	0.9900
O17—H17	0.8400	C32—H32B	0.9900
O17—C24	1.415 (6)	C33—H33A	0.9800
N1—C4	1.327 (4)	C33—H33B	0.9800
N1—C5	1.325 (5)	C33—H33C	0.9800
N2—H2	0.8800	C34—H34A	0.9900
N2—C12	1.330 (5)	C34—H34B	0.9900
N2—C13	1.343 (4)	C35—C36	1.493 (5)
N3—C20	1.330 (5)	C36—C37	1.390 (5)
N3—C21	1.333 (5)	C36—C41	1.379 (5)
N4—H4	0.8800	C37—H37	0.9500
N4—C28	1.337 (4)	C37—C38	1.375 (6)
N4—C29	1.347 (5)	C38—H38	0.9500
C1—H1A	0.9900	C38—C39	1.359 (7)
C1—H1B	0.9900	C39—H39	0.9500
C1—C2	1.500 (4)	C39—C40	1.377 (7)
C2—C3	1.402 (4)	C40—H40	0.9500
C2—C6	1.400 (5)	C40—C41	1.396 (6)
C3—C4	1.416 (4)	C41—H41	0.9500
C4—C7	1.484 (5)	O16—H16	0.8400
C5—H5	0.9500	O16—C42	1.380 (9)
C5—C6	1.373 (5)	C42—H42A	0.9900
C6—C8	1.504 (5)	C42—H42B	0.9900
C7—H7A	0.9800	C42—C43	1.384 (12)
C7—H7B	0.9800	C43—H43A	0.9800
C7—H7C	0.9800	C43—H43B	0.9800
C8—H8A	0.9900	C43—H43C	0.9800
			
Mn2—Mn1—Mn3	63.190 (13)	C28—N4—H4	118.9
O1—Mn1—Mn2	93.74 (5)	C28—N4—C29	122.1 (3)
O1—Mn1—Mn3	40.33 (4)	C29—N4—H4	118.9
O2—Mn1—Mn2	42.04 (6)	O1—C1—H1A	108.9
O2—Mn1—Mn3	34.50 (6)	O1—C1—H1B	108.9
O2—Mn1—O1	74.81 (8)	O1—C1—C2	113.5 (3)
O2—Mn1—O5	81.03 (8)	H1A—C1—H1B	107.7
O2—Mn1—O12	92.21 (9)	C2—C1—H1A	108.9
O3—Mn1—Mn2	137.18 (6)	C2—C1—H1B	108.9
O3—Mn1—Mn3	84.67 (6)	C3—C2—C1	121.5 (3)
O3—Mn1—O1	75.72 (8)	C6—C2—C1	120.2 (3)
O3—Mn1—O2	95.59 (8)	C6—C2—C3	118.2 (3)
O3—Mn1—O5	168.24 (9)	O6—C3—C2	124.1 (3)
O3—Mn1—O7	90.81 (9)	O6—C3—C4	117.3 (3)
O3—Mn1—O12	97.35 (9)	C2—C3—C4	118.6 (3)
O5—Mn1—Mn2	42.05 (6)	N1—C4—C3	121.6 (3)
O5—Mn1—Mn3	86.28 (6)	N1—C4—C7	119.3 (3)
O5—Mn1—O1	92.52 (8)	C3—C4—C7	119.1 (3)
O5—Mn1—O12	94.05 (9)	N1—C5—H5	117.9
O7—Mn1—Mn2	132.02 (6)	N1—C5—C6	124.2 (3)
O7—Mn1—Mn3	139.87 (7)	C6—C5—H5	117.9
O7—Mn1—O1	99.97 (8)	C2—C6—C8	122.7 (3)
O7—Mn1—O2	170.40 (9)	C5—C6—C2	118.3 (3)
O7—Mn1—O5	91.27 (8)	C5—C6—C8	119.0 (3)
O7—Mn1—O12	94.07 (9)	C4—C7—H7A	109.5
O12—Mn1—Mn2	81.78 (6)	C4—C7—H7B	109.5
O12—Mn1—Mn3	126.06 (6)	C4—C7—H7C	109.5
O12—Mn1—O1	164.36 (8)	H7A—C7—H7B	109.5
Mn1—Mn2—Mn3	62.784 (12)	H7A—C7—H7C	109.5
O2—Mn2—Mn1	41.74 (6)	H7B—C7—H7C	109.5
O2—Mn2—Mn3	34.32 (6)	O13—C8—C6	112.0 (4)
O2—Mn2—O4^i^	79.51 (8)	O13—C8—H8A	109.2
O2—Mn2—O11	92.14 (9)	O13—C8—H8B	109.2
O4^i^—Mn2—Mn1	98.83 (5)	C6—C8—H8A	109.2
O4^i^—Mn2—Mn3	45.19 (4)	C6—C8—H8B	109.2
O5—Mn2—Mn1	42.02 (6)	H8A—C8—H8B	107.9
O5—Mn2—Mn3	85.93 (6)	O3—C9—H9A	109.0
O5—Mn2—O2	80.72 (8)	O3—C9—H9B	109.0
O5—Mn2—O4^i^	95.70 (8)	O3—C9—C10	112.9 (3)
O5—Mn2—O11	92.82 (9)	H9A—C9—H9B	107.8
O9—Mn2—Mn1	131.22 (6)	C10—C9—H9A	109.0
O9—Mn2—Mn3	140.22 (7)	C10—C9—H9B	109.0
O9—Mn2—O2	169.68 (9)	C11—C10—C9	121.0 (3)
O9—Mn2—O4^i^	96.07 (8)	C11—C10—C14	118.9 (3)
O9—Mn2—O5	90.53 (8)	C14—C10—C9	120.1 (3)
O9—Mn2—O11	93.74 (9)	C10—C11—C15	121.1 (4)
O10—Mn2—Mn1	136.16 (6)	C12—C11—C10	119.2 (3)
O10—Mn2—Mn3	86.99 (6)	C12—C11—C15	119.7 (3)
O10—Mn2—O2	95.53 (8)	N2—C12—C11	120.3 (3)
O10—Mn2—O4^i^	76.56 (8)	N2—C12—H12	119.8
O10—Mn2—O5	171.95 (9)	C11—C12—H12	119.8
O10—Mn2—O9	92.46 (8)	N2—C13—C14	117.5 (3)
O10—Mn2—O11	94.43 (9)	N2—C13—C16	119.1 (3)
O11—Mn2—Mn1	80.99 (6)	C14—C13—C16	123.4 (3)
O11—Mn2—Mn3	125.98 (6)	O7—C14—C10	123.4 (3)
O11—Mn2—O4^i^	166.93 (8)	O7—C14—C13	116.7 (3)
Mn1^i^—Mn3—Mn1	180.0	C10—C14—C13	119.9 (3)
Mn1—Mn3—Mn2^i^	125.975 (11)	O14—C15—C11	109.7 (4)
Mn1—Mn3—Mn2	54.026 (11)	O14—C15—H15A	109.7
Mn1^i^—Mn3—Mn2	125.973 (11)	O14—C15—H15B	109.7
Mn1^i^—Mn3—Mn2^i^	54.026 (11)	C11—C15—H15A	109.7
Mn2^i^—Mn3—Mn2	180.0	C11—C15—H15B	109.7
Mn4^i^—Mn3—Mn1	116.284 (12)	H15A—C15—H15B	108.2
Mn4—Mn3—Mn1^i^	116.285 (12)	C13—C16—H16A	109.5
Mn4—Mn3—Mn1	63.716 (12)	C13—C16—H16B	109.5
Mn4^i^—Mn3—Mn1^i^	63.715 (12)	C13—C16—H16C	109.5
Mn4^i^—Mn3—Mn2	62.297 (12)	H16A—C16—H16B	109.5
Mn4—Mn3—Mn2^i^	62.298 (12)	H16A—C16—H16C	109.5
Mn4^i^—Mn3—Mn2^i^	117.702 (12)	H16B—C16—H16C	109.5
Mn4—Mn3—Mn2	117.703 (12)	O4—C17—H17A	108.3
Mn4^i^—Mn3—Mn4	180.0	O4—C17—H17B	108.3
O1^i^—Mn3—Mn1^i^	51.20 (6)	O4—C17—C18	115.8 (3)
O1—Mn3—Mn1^i^	128.80 (6)	H17A—C17—H17B	107.4
O1^i^—Mn3—Mn1	128.80 (6)	C18—C17—H17A	108.3
O1—Mn3—Mn1	51.20 (6)	C18—C17—H17B	108.3
O1^i^—Mn3—Mn2^i^	94.51 (6)	C19—C18—C17	122.2 (3)
O1—Mn3—Mn2	94.51 (6)	C19—C18—C22	118.0 (3)
O1^i^—Mn3—Mn2	85.49 (6)	C22—C18—C17	119.8 (3)
O1—Mn3—Mn2^i^	85.49 (6)	O8—C19—C18	125.0 (3)
O1—Mn3—Mn4^i^	142.37 (5)	O8—C19—C20	116.1 (3)
O1—Mn3—Mn4	37.63 (5)	C18—C19—C20	118.9 (3)
O1^i^—Mn3—Mn4^i^	37.64 (5)	N3—C20—C19	121.4 (3)
O1^i^—Mn3—Mn4	142.37 (5)	N3—C20—C23	118.3 (3)
O1^i^—Mn3—O1	180.0	C19—C20—C23	120.3 (3)
O1—Mn3—O4^i^	104.56 (7)	N3—C21—H21	118.1
O1^i^—Mn3—O4^i^	75.44 (7)	N3—C21—C22	123.8 (4)
O1^i^—Mn3—O4	104.56 (7)	C22—C21—H21	118.1
O1—Mn3—O4	75.44 (7)	C18—C22—C24	122.5 (3)
O2^i^—Mn3—Mn1^i^	34.74 (6)	C21—C22—C18	118.7 (3)
O2^i^—Mn3—Mn1	145.26 (6)	C21—C22—C24	118.8 (3)
O2—Mn3—Mn1	34.74 (6)	C20—C23—H23A	109.5
O2—Mn3—Mn1^i^	145.26 (6)	C20—C23—H23B	109.5
O2^i^—Mn3—Mn2	145.20 (6)	C20—C23—H23C	109.5
O2^i^—Mn3—Mn2^i^	34.80 (6)	H23A—C23—H23B	109.5
O2—Mn3—Mn2	34.80 (6)	H23A—C23—H23C	109.5
O2—Mn3—Mn2^i^	145.21 (6)	H23B—C23—H23C	109.5
O2—Mn3—Mn4^i^	88.58 (6)	O17—C24—C22	110.3 (4)
O2—Mn3—Mn4	91.42 (6)	O17—C24—H24A	109.6
O2^i^—Mn3—Mn4^i^	91.42 (6)	O17—C24—H24B	109.6
O2^i^—Mn3—Mn4	88.58 (6)	C22—C24—H24A	109.6
O2—Mn3—O1	85.92 (8)	C22—C24—H24B	109.6
O2—Mn3—O1^i^	94.08 (8)	H24A—C24—H24B	108.1
O2^i^—Mn3—O1^i^	85.92 (8)	O5—C25—H25A	109.3
O2^i^—Mn3—O1	94.08 (8)	O5—C25—H25B	109.3
O2^i^—Mn3—O2	180.00 (12)	O5—C25—C26	111.8 (3)
O2—Mn3—O4	96.56 (8)	H25A—C25—H25B	107.9
O2—Mn3—O4^i^	83.44 (8)	C26—C25—H25A	109.3
O2^i^—Mn3—O4	83.45 (8)	C26—C25—H25B	109.3
O2^i^—Mn3—O4^i^	96.55 (8)	C25—C26—H26A	109.5
O4^i^—Mn3—Mn1	94.02 (5)	C25—C26—H26B	109.5
O4—Mn3—Mn1^i^	94.02 (5)	C25—C26—H26C	109.5
O4—Mn3—Mn1	85.98 (5)	H26A—C26—H26B	109.5
O4^i^—Mn3—Mn1^i^	85.97 (5)	H26A—C26—H26C	109.5
O4—Mn3—Mn2^i^	48.66 (5)	H26B—C26—H26C	109.5
O4—Mn3—Mn2	131.34 (5)	O9—C27—C28	115.6 (3)
O4^i^—Mn3—Mn2	48.66 (5)	O9—C27—C31	124.3 (3)
O4^i^—Mn3—Mn2^i^	131.34 (5)	C31—C27—C28	120.1 (3)
O4^i^—Mn3—Mn4^i^	37.81 (5)	N4—C28—C27	119.1 (3)
O4—Mn3—Mn4^i^	142.19 (5)	N4—C28—C33	118.8 (3)
O4^i^—Mn3—Mn4	142.19 (5)	C27—C28—C33	122.1 (3)
O4—Mn3—Mn4	37.81 (5)	N4—C29—H29	119.5
O4^i^—Mn3—O4	180.0	N4—C29—C30	120.9 (3)
O1—Mn4—Mn3	39.83 (6)	C30—C29—H29	119.5
O1—Mn4—O3	80.23 (8)	C29—C30—C31	119.4 (3)
O1—Mn4—O10^i^	88.94 (8)	C29—C30—C34	119.9 (3)
O3—Mn4—Mn3	80.49 (5)	C31—C30—C34	120.7 (3)
O4—Mn4—Mn3	45.98 (5)	C27—C31—C30	118.3 (3)
O4—Mn4—O1	85.80 (8)	C27—C31—C32	121.1 (3)
O4—Mn4—O3	86.40 (8)	C30—C31—C32	120.6 (3)
O4—Mn4—O10^i^	80.29 (8)	O10—C32—C31	114.1 (2)
O6—Mn4—Mn3	131.65 (6)	O10—C32—H32A	108.7
O6—Mn4—O1	92.27 (9)	O10—C32—H32B	108.7
O6—Mn4—O3	101.04 (9)	C31—C32—H32A	108.7
O6—Mn4—O4	171.90 (10)	C31—C32—H32B	108.7
O6—Mn4—O8	89.03 (9)	H32A—C32—H32B	107.6
O6—Mn4—O10^i^	91.82 (9)	C28—C33—H33A	109.5
O8—Mn4—Mn3	139.31 (7)	C28—C33—H33B	109.5
O8—Mn4—O1	172.24 (10)	C28—C33—H33C	109.5
O8—Mn4—O3	92.02 (9)	H33A—C33—H33B	109.5
O8—Mn4—O4	93.95 (9)	H33A—C33—H33C	109.5
O8—Mn4—O10^i^	98.67 (9)	H33B—C33—H33C	109.5
O10^i^—Mn4—Mn3	83.17 (5)	O15—C34—C30	109.8 (3)
O10^i^—Mn4—O3	163.43 (7)	O15—C34—H34A	109.7
Mn3—O1—Mn1	88.47 (7)	O15—C34—H34B	109.7
Mn4—O1—Mn1	96.62 (8)	C30—C34—H34A	109.7
Mn4—O1—Mn3	102.54 (8)	C30—C34—H34B	109.7
C1—O1—Mn1	124.55 (19)	H34A—C34—H34B	108.2
C1—O1—Mn3	120.65 (17)	O11—C35—O12	125.0 (3)
C1—O1—Mn4	117.86 (17)	O11—C35—C36	117.6 (3)
Mn1—O2—Mn2	96.23 (8)	O12—C35—C36	117.5 (3)
Mn3—O2—Mn1	110.77 (10)	C37—C36—C35	120.2 (3)
Mn3—O2—Mn2	110.88 (10)	C41—C36—C35	120.9 (3)
Mn1—O3—Mn4	107.22 (9)	C41—C36—C37	118.9 (4)
C9—O3—Mn1	117.61 (19)	C36—C37—H37	119.6
C9—O3—Mn4	121.6 (2)	C38—C37—C36	120.7 (4)
Mn3—O4—Mn2^i^	86.15 (7)	C38—C37—H37	119.6
Mn4—O4—Mn2^i^	97.47 (8)	C37—C38—H38	119.8
Mn4—O4—Mn3	96.21 (8)	C39—C38—C37	120.5 (5)
C17—O4—Mn2^i^	123.30 (17)	C39—C38—H38	119.8
C17—O4—Mn3	125.88 (16)	C38—C39—H39	120.1
C17—O4—Mn4	119.61 (17)	C38—C39—C40	119.8 (5)
Mn1—O5—Mn2	95.94 (8)	C40—C39—H39	120.1
C25—O5—Mn1	120.45 (18)	C39—C40—H40	119.8
C25—O5—Mn2	120.49 (17)	C39—C40—C41	120.4 (5)
C3—O6—Mn4	128.97 (18)	C41—C40—H40	119.8
C14—O7—Mn1	128.61 (19)	C36—C41—C40	119.6 (4)
C19—O8—Mn4	127.91 (18)	C36—C41—H41	120.2
C27—O9—Mn2	127.82 (18)	C40—C41—H41	120.2
Mn2—O10—Mn4^i^	105.48 (9)	C42—O16—H16	109.5
C32—O10—Mn2	119.09 (18)	O16—C42—H42A	110.8
C32—O10—Mn4^i^	120.87 (19)	O16—C42—H42B	110.8
C35—O11—Mn2	127.0 (2)	O16—C42—C43	104.9 (8)
C35—O12—Mn1	125.1 (2)	H42A—C42—H42B	108.8
C8—O13—H13	109.5	C43—C42—H42A	110.8
C15—O14—H14	109.5	C43—C42—H42B	110.8
C34—O15—H15	109.5	C42—C43—H43A	109.5
C24—O17—H17	109.5	C42—C43—H43B	109.5
C5—N1—C4	119.1 (3)	C42—C43—H43C	109.5
C12—N2—H2	117.9	H43A—C43—H43B	109.5
C12—N2—C13	124.1 (3)	H43A—C43—H43C	109.5
C13—N2—H2	117.9	H43B—C43—H43C	109.5
C20—N3—C21	119.2 (3)		
			
Mn1—Mn2—O9—C27	166.9 (2)	O11—C35—C36—C41	177.9 (3)
Mn1—Mn2—O10—Mn4^i^	85.65 (11)	O12—Mn1—O3—Mn4	−169.09 (9)
Mn1—Mn2—O10—C32	−134.58 (19)	O12—Mn1—O3—C9	49.5 (2)
Mn1—O1—C1—C2	−64.4 (3)	O12—Mn1—O7—C14	−89.6 (3)
Mn1—O3—C9—C10	63.5 (3)	O12—C35—C36—C37	178.2 (3)
Mn1—O5—C25—C26	−127.1 (3)	O12—C35—C36—C41	−2.5 (5)
Mn1—O7—C14—C10	12.5 (5)	N1—C5—C6—C2	−0.1 (7)
Mn1—O7—C14—C13	−167.8 (2)	N1—C5—C6—C8	178.7 (4)
Mn1—O12—C35—O11	3.5 (4)	N2—C13—C14—O7	−178.4 (3)
Mn1—O12—C35—C36	−176.0 (2)	N2—C13—C14—C10	1.3 (5)
Mn2—Mn1—O3—Mn4	−83.27 (12)	N3—C21—C22—C18	0.6 (7)
Mn2—Mn1—O3—C9	135.3 (2)	N3—C21—C22—C24	179.5 (4)
Mn2—Mn1—O7—C14	−172.2 (2)	N4—C29—C30—C31	−1.5 (6)
Mn2^i^—O4—C17—C18	−74.6 (3)	N4—C29—C30—C34	179.7 (4)
Mn2—O5—C25—C26	113.8 (3)	C1—C2—C3—O6	4.7 (5)
Mn2—O9—C27—C28	171.4 (2)	C1—C2—C3—C4	−173.4 (3)
Mn2—O9—C27—C31	−8.0 (5)	C1—C2—C6—C5	174.9 (4)
Mn2—O10—C32—C31	−57.1 (3)	C1—C2—C6—C8	−3.8 (6)
Mn2—O11—C35—O12	−5.1 (5)	C2—C3—C4—N1	−3.1 (5)
Mn2—O11—C35—C36	174.4 (2)	C2—C3—C4—C7	176.2 (3)
Mn3—Mn1—O3—Mn4	−43.35 (7)	C2—C6—C8—O13	71.6 (5)
Mn3—Mn1—O3—C9	175.2 (2)	C3—C2—C6—C5	−1.9 (6)
Mn3—Mn1—O7—C14	90.5 (3)	C3—C2—C6—C8	179.4 (4)
Mn3—Mn2—O9—C27	−97.2 (2)	C4—N1—C5—C6	0.5 (7)
Mn3—Mn2—O10—Mn4^i^	41.38 (7)	C5—N1—C4—C3	1.1 (6)
Mn3—Mn2—O10—C32	−178.9 (2)	C5—N1—C4—C7	−178.2 (4)
Mn3—Mn4—O6—C3	−2.7 (3)	C5—C6—C8—O13	−107.1 (5)
Mn3—Mn4—O8—C19	−2.2 (3)	C6—C2—C3—O6	−178.5 (3)
Mn3—O1—C1—C2	−176.10 (19)	C6—C2—C3—C4	3.4 (5)
Mn3—O4—C17—C18	173.3 (2)	C9—C10—C11—C12	−179.9 (4)
Mn4—O1—C1—C2	57.1 (3)	C9—C10—C11—C15	−0.6 (6)
Mn4—O3—C9—C10	−72.1 (3)	C9—C10—C14—O7	−0.6 (5)
Mn4—O4—C17—C18	48.9 (3)	C9—C10—C14—C13	179.7 (3)
Mn4—O6—C3—C2	11.1 (5)	C10—C11—C12—N2	−0.9 (6)
Mn4—O6—C3—C4	−170.8 (2)	C10—C11—C15—O14	169.2 (4)
Mn4—O8—C19—C18	7.3 (5)	C11—C10—C14—O7	177.6 (3)
Mn4—O8—C19—C20	−173.6 (2)	C11—C10—C14—C13	−2.1 (5)
Mn4^i^—O10—C32—C31	76.4 (3)	C12—N2—C13—C14	−0.3 (5)
O1—Mn1—O3—Mn4	−3.38 (7)	C12—N2—C13—C16	−178.4 (4)
O1—Mn1—O3—C9	−144.8 (2)	C12—C11—C15—O14	−11.5 (6)
O1—Mn1—O7—C14	83.5 (3)	C13—N2—C12—C11	0.0 (6)
O1—Mn4—O6—C3	3.8 (3)	C14—C10—C11—C12	1.9 (6)
O1—C1—C2—C3	−40.1 (4)	C14—C10—C11—C15	−178.8 (4)
O1—C1—C2—C6	143.2 (3)	C15—C11—C12—N2	179.8 (4)
O2—Mn1—O3—Mn4	−76.12 (10)	C16—C13—C14—O7	−0.4 (5)
O2—Mn1—O3—C9	142.4 (2)	C16—C13—C14—C10	179.3 (3)
O2—Mn2—O9—C27	−149.7 (5)	C17—C18—C19—O8	2.5 (5)
O2—Mn2—O10—Mn4^i^	74.66 (10)	C17—C18—C19—C20	−176.5 (3)
O2—Mn2—O10—C32	−145.6 (2)	C17—C18—C22—C21	176.8 (4)
O3—Mn1—O7—C14	7.8 (3)	C17—C18—C22—C24	−2.0 (6)
O3—Mn4—O6—C3	84.3 (3)	C18—C19—C20—N3	−1.0 (5)
O3—Mn4—O8—C19	−80.0 (3)	C18—C19—C20—C23	178.8 (3)
O3—C9—C10—C11	144.1 (3)	C18—C22—C24—O17	64.0 (5)
O3—C9—C10—C14	−37.7 (5)	C19—C18—C22—C21	−0.2 (6)
O4^i^—Mn2—O9—C27	−85.7 (3)	C19—C18—C22—C24	−179.1 (4)
O4^i^—Mn2—O10—Mn4^i^	−3.14 (7)	C20—N3—C21—C22	−1.1 (7)
O4^i^—Mn2—O10—C32	136.6 (2)	C21—N3—C20—C19	1.2 (6)
O4—Mn4—O8—C19	6.6 (3)	C21—N3—C20—C23	−178.5 (4)
O4—C17—C18—C19	−32.1 (5)	C21—C22—C24—O17	−114.8 (4)
O4—C17—C18—C22	151.0 (3)	C22—C18—C19—O8	179.5 (3)
O5—Mn1—O3—Mn4	−3.5 (5)	C22—C18—C19—C20	0.4 (5)
O5—Mn1—O3—C9	−144.9 (4)	C27—C31—C32—O10	35.1 (4)
O5—Mn1—O7—C14	176.2 (3)	C28—N4—C29—C30	1.9 (6)
O5—Mn2—O9—C27	178.5 (3)	C28—C27—C31—C30	1.4 (5)
O6—Mn4—O8—C19	179.0 (3)	C28—C27—C31—C32	178.7 (3)
O6—C3—C4—N1	178.7 (3)	C29—N4—C28—C27	−0.6 (5)
O6—C3—C4—C7	−2.0 (5)	C29—N4—C28—C33	179.2 (4)
O7—Mn1—O3—Mn4	96.70 (10)	C29—C30—C31—C27	−0.1 (5)
O7—Mn1—O3—C9	−44.7 (2)	C29—C30—C31—C32	−177.5 (4)
O8—Mn4—O6—C3	176.1 (3)	C29—C30—C34—O15	7.5 (6)
O8—C19—C20—N3	179.9 (3)	C30—C31—C32—O10	−147.7 (3)
O8—C19—C20—C23	−0.4 (5)	C31—C27—C28—N4	−1.1 (5)
O9—Mn2—O10—Mn4^i^	−98.80 (10)	C31—C27—C28—C33	179.1 (3)
O9—Mn2—O10—C32	41.0 (2)	C31—C30—C34—O15	−171.2 (4)
O9—C27—C28—N4	179.5 (3)	C34—C30—C31—C27	178.6 (4)
O9—C27—C28—C33	−0.4 (5)	C34—C30—C31—C32	1.3 (5)
O9—C27—C31—C30	−179.2 (3)	C35—C36—C37—C38	179.5 (3)
O9—C27—C31—C32	−1.9 (5)	C35—C36—C41—C40	−179.0 (3)
O10—Mn2—O9—C27	−9.0 (3)	C36—C37—C38—C39	−0.3 (6)
O10^i^—Mn4—O6—C3	−85.2 (3)	C37—C36—C41—C40	0.3 (5)
O10^i^—Mn4—O8—C19	87.3 (3)	C37—C38—C39—C40	−0.1 (7)
O11—Mn2—O9—C27	85.6 (3)	C38—C39—C40—C41	0.6 (7)
O11—Mn2—O10—Mn4^i^	167.25 (9)	C39—C40—C41—C36	−0.7 (6)
O11—Mn2—O10—C32	−53.0 (2)	C41—C36—C37—C38	0.2 (5)
O11—C35—C36—C37	−1.4 (5)		

Symmetry code: (i) −*x*+1, −*y*+1, −*z*+1.

### Hydrogen-bond geometry (Å, º)

**Table:**

*D*—H···*A*	*D*—H	H···*A*	*D*···*A*	*D*—H···*A*
O14—H14···N3^ii^	0.84	1.80	2.619 (4)	163
O15—H15···N1^iii^	0.84	1.84	2.675 (4)	175
O17—H17···O13^i^	0.84	1.89	2.684 (5)	158

Symmetry codes: (i) −*x*+1, −*y*+1, −*z*+1; (ii) −*x*+1, −*y*, −*z*+2; (iii) *x*, *y*, *z*−1.
